# The interstitium at the developing nephron in the fetal kidney during advanced pregnancy — a microanatomical inventory

**DOI:** 10.1186/s40348-022-00149-9

**Published:** 2022-08-26

**Authors:** Will W. Minuth

**Affiliations:** Institute of Anatomy, University of Regensburg, 93053 Regensburg, Germany

**Keywords:** Fetal human kidney, Nephrogenic zone, Nephron development, Interstitium, Stroma, Impairment of nephrogenesis

## Abstract

**Background:**

A series of noxae can evoke the termination of nephron formation in preterm and low birth weight babies. This results in oligonephropathy with severe consequences for health in the later life. Although the clinical parameters have been extensively investigated, little is known about the initial damage. Previous pathological findings indicate the reduction in width of the nephrogenic zone and the lack of S-shaped bodies. Current morphological investigations suggest that due to the mutual patterning beside the forming nephron, also its structural neighbors, particularly the interjacent interstitium, must be affected. However, beside the findings on integrative and mastering functions, systematic microanatomical data explaining the configuration of the interstitium at the developing nephron in the fetal kidney during advanced pregnancy is not available. Therefore, this work explains the typical features.

**Results:**

The generated data depicts that the progenitor cells, nephrogenic niche, pretubular aggregate, and mesenchymal-to-epithelial transition are restricted to the subcapsular interstitium. During the proceeding development, only the distal pole of the renal vesicles and comma- and S-shaped bodies stays in further contact with it. The respective proximal pole is positioned opposite the peritubular interstitium at the connecting tubule of an underlying but previously formed nephron. The related medial aspect faces the narrow peritubular interstitium of a collecting duct (CD) ampulla first only at its tip, then at its head, conus, and neck, and finally at the differentiating CD tubule. The lateral aspect starts at the subcapsular interstitium, but then it is positioned along the wide perivascular interstitium of the neighboring ascending perforating radiate artery. When the nephron matures, the interstitial configuration changes again.

**Conclusions:**

The present investigation illustrates that the interstitium at the forming nephron in the fetal kidney consists of existing, transient, stage-specific, and differently far matured compartments. According to the developmental needs, it changes its shape by formation, degradation, fusion, and rebuilding.

## Background

The clinical experiences with preterm and low birth weight babies demonstrate that the developing kidneys are particularly vulnerable from mid to late pregnancy. As well as malnutrition, diabetes and vitamin deficiency of the mother as insufficiency of the placenta, along with hyperoxia and drugs, can evoke the termination of nephron formation, which leads to severe consequences for health in the later life [1–4]. Pathological findings indicate that the noxae target the outer cortex of the fetal kidney. In its external nephrogenic zone, the reduction of its vertical width [5] and the loss of here occurring S-shaped bodies [6] were registered. In the subjacent maturation zone, a reduced number of glomeruli and the rise of atypical glomeruli with an extended Bowman’s space and a shrunken glomerular tuft were observed [7].

The reduction of the nephrogenic zone [5] and the lack of S-shaped bodies [6] in the fetal human kidney are signs that the early links in the chain of nephron formation are broken. Although these massive pathological alterations have been detected, it is currently unknown, whether the neighboring structures such as the earlier stages of nephron anlage, the renal capsule covering, the stock of mesenchymal progenitor cells, the ureteric bud derived collecting duct ampullae, and the interstitium including the sprouting of microvessels are collaterally affected. However, it is of great importance to have a clear understanding of the intactness and the respective damage, since the development of a nephron is not an isolated and autonomously running process. In fact, it depends on intact tissue and cell-to-cell interactions [8, 9] such as the spatial and temporal exchange of morphogenic proteins [10, 11] and the mutual patterning with its structural neighbors [12].

Little consideration for the progress of nephron development has received the establishing stroma (Greek) or even called interstitium (Latin). It directs not only the interplay between the ureteric bud-derived tips at the collecting duct tubules and the surrounding nephrogenic mesenchymal progenitor cells during the process of induction but also various subsequent molecular interactions [13, 14]. For the rodent kidney, it was shown that the ablation of the FOXD1^+^ progenitor cells leads to a reduction of branching at the terminal tips of the collecting duct tubules, an uncontrolled expansion of the progenitor cells, an abnormal development of the renal capsule, and a severe alteration in the vascular patterning, which results in a reduced number of nephrons [15, 16]. Furthermore, it was demonstrated that the disturbed microRNA biogenesis in FOXD1^+^ progenitor cells by the loss of Dicer activity causes multifaceted abnormalities in the developing nephron [17].

Regarding the interstitium as a mastering instance, it requires special attention, when the initial traces left by the noxae impairing nephrogenesis are investigated in the kidneys of preterm and low birth weight babies. However, it has not been explored by which cell biological mechanism the FOXD1^+^ progenitor cells control the process of nephron formation and at which cellular or molecular level the noxae show an interference with it. Most surprisingly, the screening of relevant literature revealed that neither data dealing with the precise location of the FOXD1^+^ progenitor cells at the transient stages of nephron anlage nor data about the expansion of the interstitium at the forming nephron in the fetal human kidney during advanced pregnancy is available. Therefore, to support the search for initial traces left by the impairment of nephrogenesis, an inventory of the interstitium at the forming nephron, seen from a microanatomical perspective, was made.

## Material and methods

To obtain a systematic morphological picture about the interstitium at the forming nephron, it is recommended to follow the successive stages of nephron anlage such as the nephrogenic niche, pretubular aggregate, renal vesicles, comma- and S-shaped bodies, and the maturing nephron. In a fetal kidney during advanced pregnancy, the formation of a nephron starts in the nephrogenic zone, which extends along the entire inner side of the renal capsule. Damage during the histological preparation of this particular region must be prevented. For this reason, a fetal kidney is held only on the hilum, and the touching of the renal capsule with fine forceps has to be avoided. Further on, for obtaining comparable perspectives in the histological sections, a fixed kidney is cut from the renal capsule towards the papilla of a lobe. Following this, the section plane lines along the axis of vertically running collecting duct (CD) tubules and perpendicular to the renal capsule [18].

For the shown illustrations, specimens of the fetal human kidneys are of gestational age between week 16, 18, and later. They were selected from the stock of preparations used for the course of microscopic anatomy for medical students at the University of Regensburg in Germany. These stages are insofar of special interest, because up until birth, the majority of nephrons is formed.

According to routine methods, the tissue blocks were preserved in paraformaldehyde solution and embedded in paraffin wax. Then, sections of 5 µm thickness were cut and stained with hematoxylin–eosin solution for analysis by an optical microscope. Screening of the stained sections was performed by a Leica DM750 microscope (Leica Microsystems, Wetzlar, Germany). The stages of nephron anlage were analyzed with a HI Plan 63 × /0.75 objective lens. Images were taken with a Basler microscopy pulse 5.0 camera (Basler AG, Ahrensburg, Germany). The analyzed specimens had a normal anatomical look and were of good histological preservation. Renal cysts or other pathological alterations were not visible. Each of the selected images in the shown illustrations was selected as the most suitable from a series of at least 5 comparable specimens.

More than 3000 images were available, which had been analyzed for an earlier performed investigation dealing with the shaping of the nephron [19, 20]. From this stock, those representative images were selected, which illustrate the basic stages of initial nephron formation including the expansion of the related interstitium. This is located in the space between the individual stages of nephron anlage and the respective covering tissues. The different sites of the expanding interstitium were allocated to specific structures (Table 1) and labeled by individual symbols (Table 2).

**Table 1:**
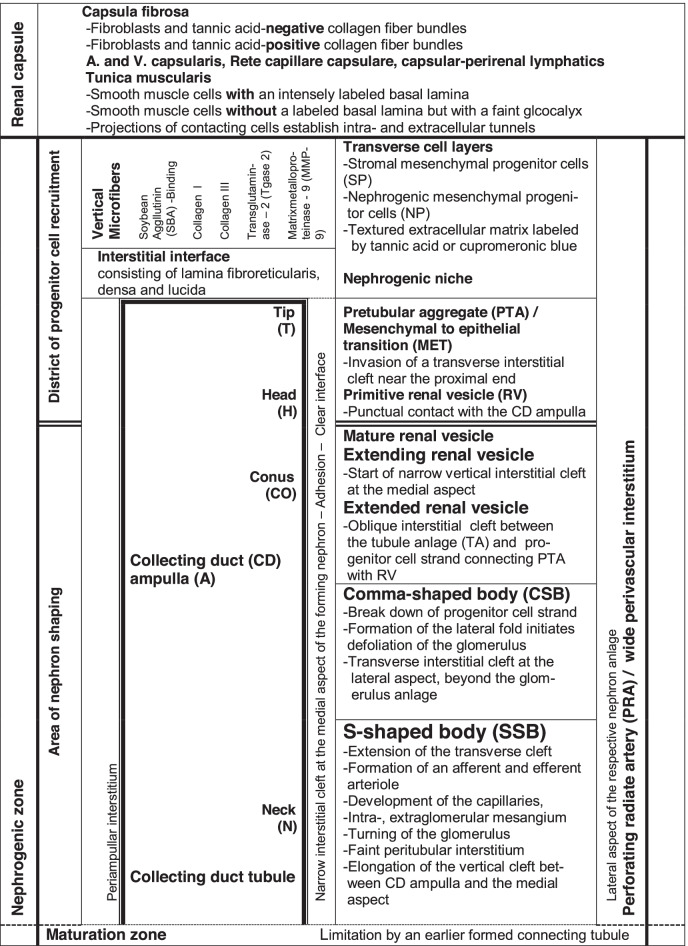
List of interstitial sites at the forming nephron in the fetal human kidney during advanced pregnancy. The nephrogenic compartment is marked by a thick black line, the collecting duct (CD) ampulla by a thick/thin line. The transverse double line separates the district of progenitor cell recruitment from the area of nephron shaping

**Table 2:**
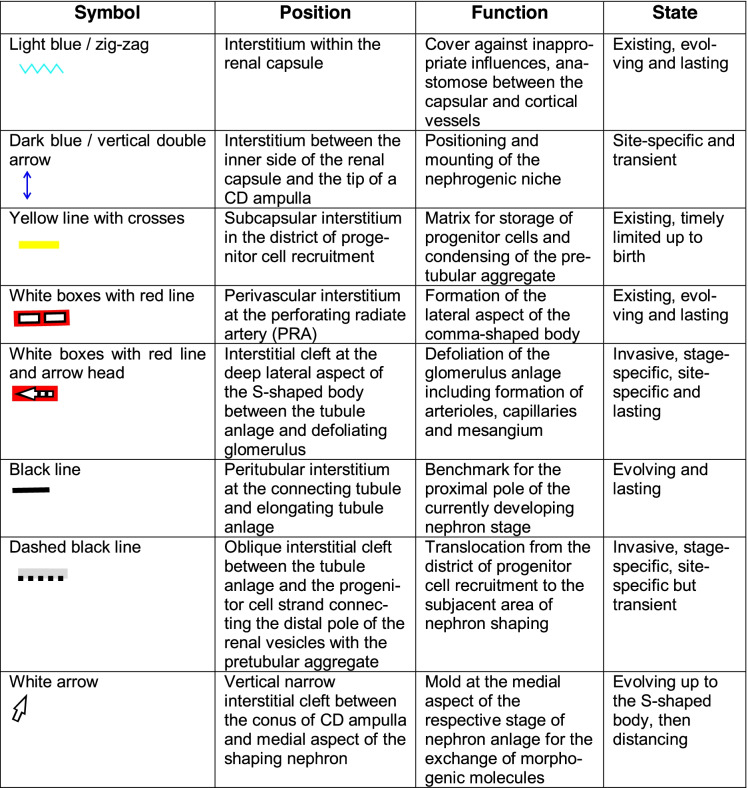
List of symbols, position, and possible functions of the interstitium at the developing nephron in the fetal human kidney during advanced pregnancy

In order to depict the temporal and spatial alterations of the interstitium, beside the photographic illustrations, a series of graphical sketches almost true to scale were produced. To meet the defined criteria, the borders between the individual stages of nephron anlage and their covering tissues were marked 1:1 by hand. In a further step, the generated contours of the interstitium were scanned, edited, and processed by the design program CorelDRAW X7 (Corel Corporation, Munich, Germany). The same program was used for inserting the necessary labels and for obtaining information about the metric parameters in the microscopic images or produced sketches. Finally, to visualize the interstitial changes, mounted image plates were produced. As it is indicated, the proximal pole (fixed point) of the currently forming stage of nephron anlage rests next to the connecting tubule of a previously developed nephron. Since the space for development is occupied at the proximal pole, its distal pole (mobile point) shifts while the expansion is in the vertical direction. Thereby, it postpones radially the overlying structures including the pool of progenitor cells and the renal capsule.

To illustrate the individual structural features of the interstitium between the inner side of the renal capsule and the tip of the CD ampulla, immunohistochemical labeling on sections of neonatal rabbit kidney was performed as it was earlier described [18]. For the present investigations, the cryosections were incubated with monoclonal anti-collagen type 3 antibody (Calbiochem, Schwalbach, Germany). The same specimens were used to demonstrate the ultrastructure of the interface at the CD ampulla tip. The special fixation and contrasting for the analysis in the transmission electron microscopy have been performed by 5% glutaraldehyde buffered with 0.15 M sodium cacodylate, pH 7.4 containing additionally either 1% tannic acid (Sigma-Aldrich Chemie, München, Germany), or 0.1% cupromeronic blue (Santa Cruz, Heidelberg, Germany).

## Results

### Interstitium as an integrating element

**Fig. 1:**
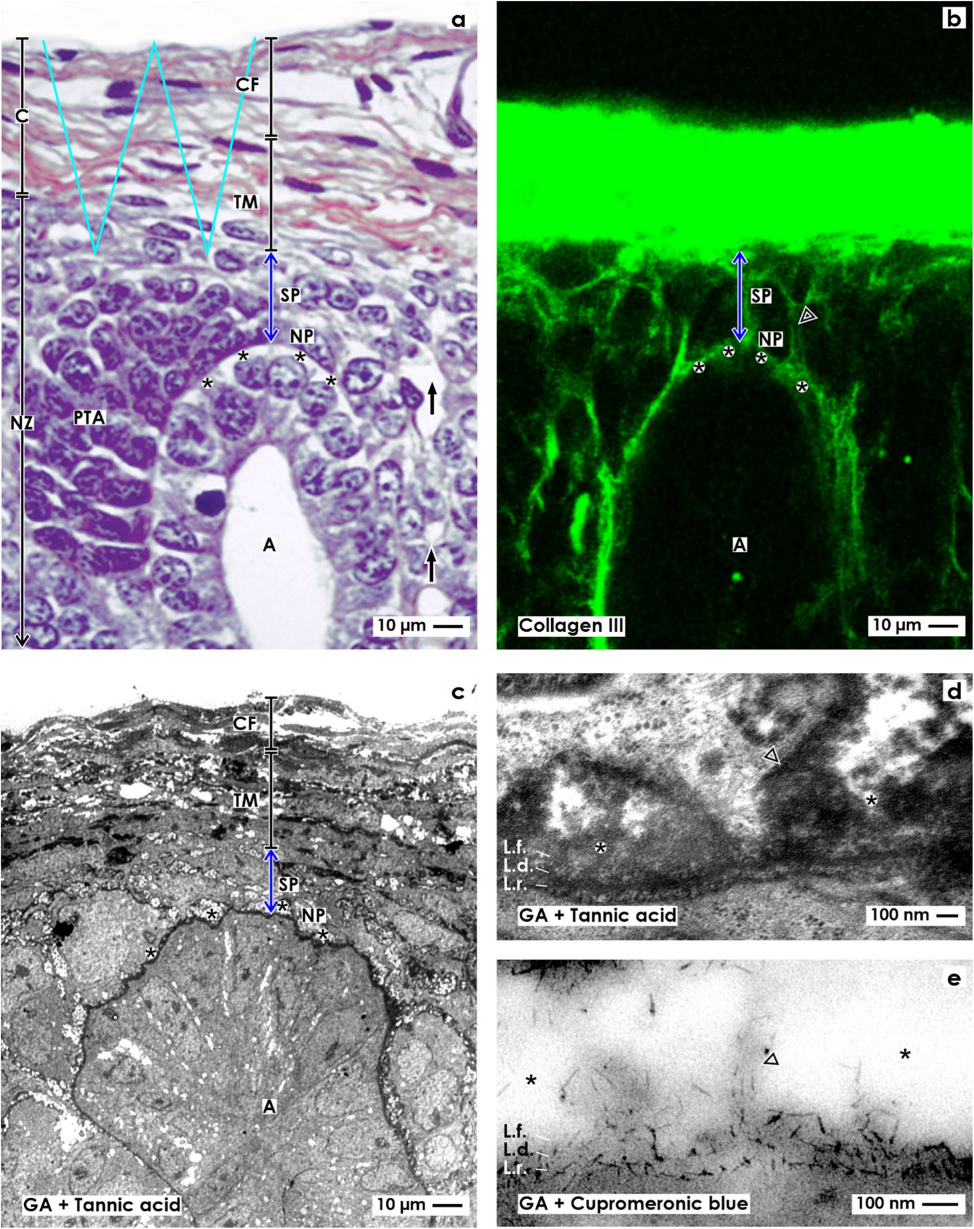
**a**–**e** View onto the renal capsule (C light blue/zigzag) and the external part of the nephrogenic zone (NZ) in the **a** fetal human kidney during advanced pregnancy and **b**–**e** the neonatal rabbit kidney. **a** By the optical microscope, one can recognize in the external capsula fibrosa (CF) arterial, venous, and lymphatic vessels between the bundles of collagen. In the subjacent tunica muscularis (TM), smooth muscle cells are seen. Between the inner side of the renal capsule and the tip of a CD ampulla (A dark blue/vertical double arrow), a transverse outer layer of interstitial/stromal (SP) and 2 to 3 inner layers of nephrogenic (NP) progenitor cells are present. The innermost layer is separated by a clear interface (black asterisks) from the subjacent tip of a collecting duct (CD) ampulla (A). **b** Immunohistochemical label for collagen type 3 depicts that the tip of a CD ampulla is linked via microfibers (arrow head) with the inner side of the renal capsule. **c** Contrasting by tannic acid for transmission electron microscopy illustrates a pronounced label at the interface, which surrounds the tip of a CD ampulla. Higher magnification of **d** tannic acid or **e** cupromeronic blue contrasted specimens indicates that textured extracellular matrix is contained at the lamina rara (Lr), densa (Ld), and fibroreticularis (Lf) of the here occurring basal lamina. PTA, pretubular aggregate (black arrow, PRA), perforating radiate artery

**Fig. 2:**
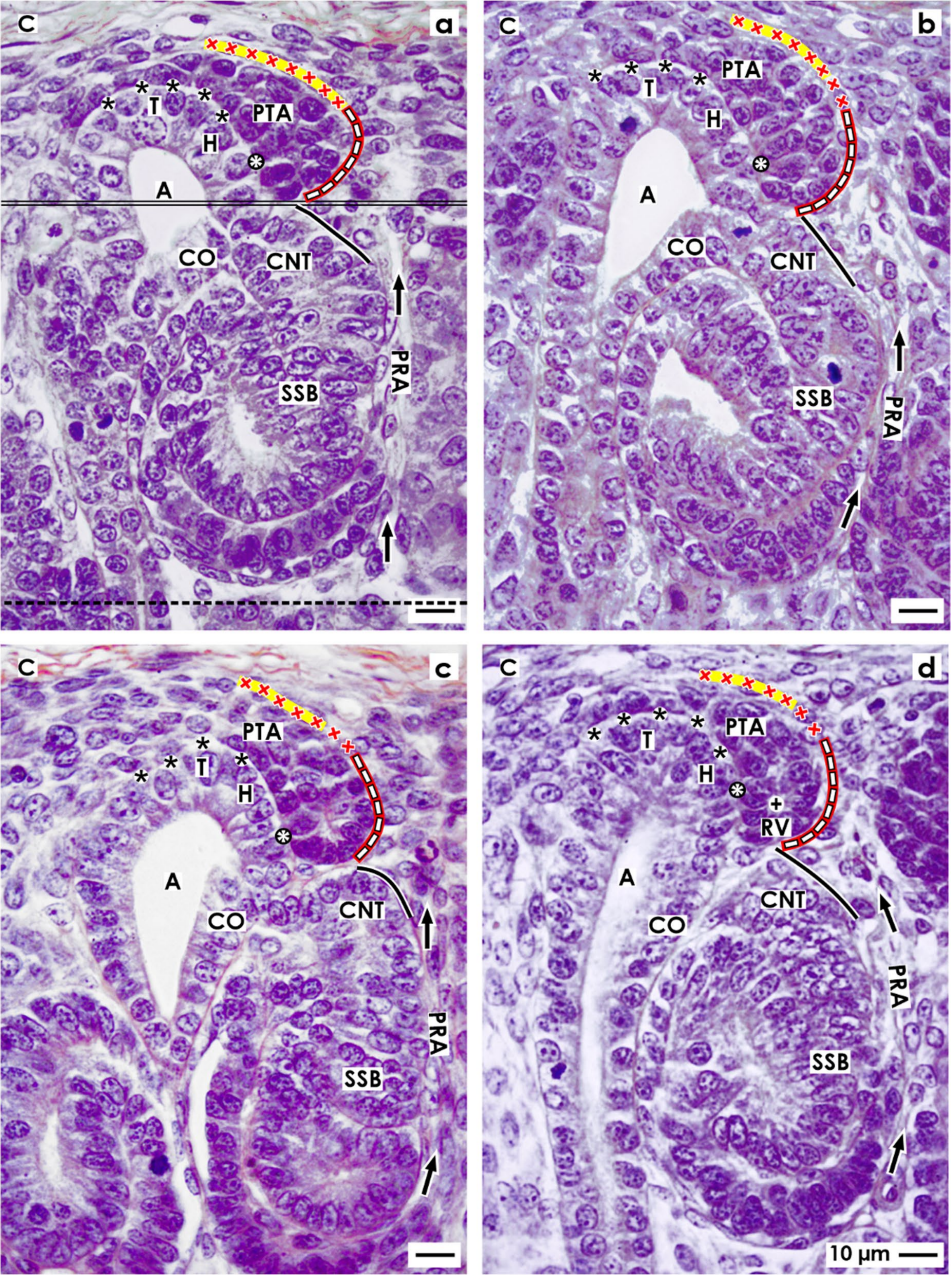
**a**–**d** View onto the interstitial situation at the **a** nephrogenic niche and pretubular aggregate (PTA), **b** mesenchymal-to-epithelial transition, **c** arising, and **d** established primitive renal vesicle (RV) in the fetal human kidney during advanced pregnancy by the optical microscope. The transverse double line marks the border between the district of progenitor cell recruitment and the underlying area of nephron shaping. The dashed line indicates the border between the overlying nephrogenic zone and the subjacent maturation zone. **a** At the niche site, the nephrogenic progenitor cells are separated by a clear interface (black asterisks) from the epithelial progenitor cells, which are contained in the tip (T) of a CD ampulla (A). **b** During the mesenchymal-to-epithelial transition, the medial part of the PTA faces the clear interface, while its lateral part is exposed to the subcapsular interstitium (yellow line with crosses). Determining, the medial side at the proximal end of the PTA shows an adhesion (white asterisk) to the CD ampulla at the section border between its head (H) and conus (CO). The mid of the proximal end at the PTA faces the interstitium at the connecting tubule (black line) of a previously developed nephron. The lateral side of the proximal end at the PTA is positioned near the perivascular interstitium (white boxes with red line) of an ascending perforating radiate artery (PRA). **c** and **d** The arising primitive RV presents polarized epithelial cells first at the proximal end of the PTA. Here, also a small lumen ( +) becomes visible. The distal pole of the RV remains preliminarily connected with the PTA. The basal lamina at the proximal pole of the RV is part of a cone, which is formed by the perivascular interstitium of the PRA and the peritubular interstitium of the connecting tubule (CNT) at a previously formed nephron. SSB, S-shaped body

**Fig. 3:**
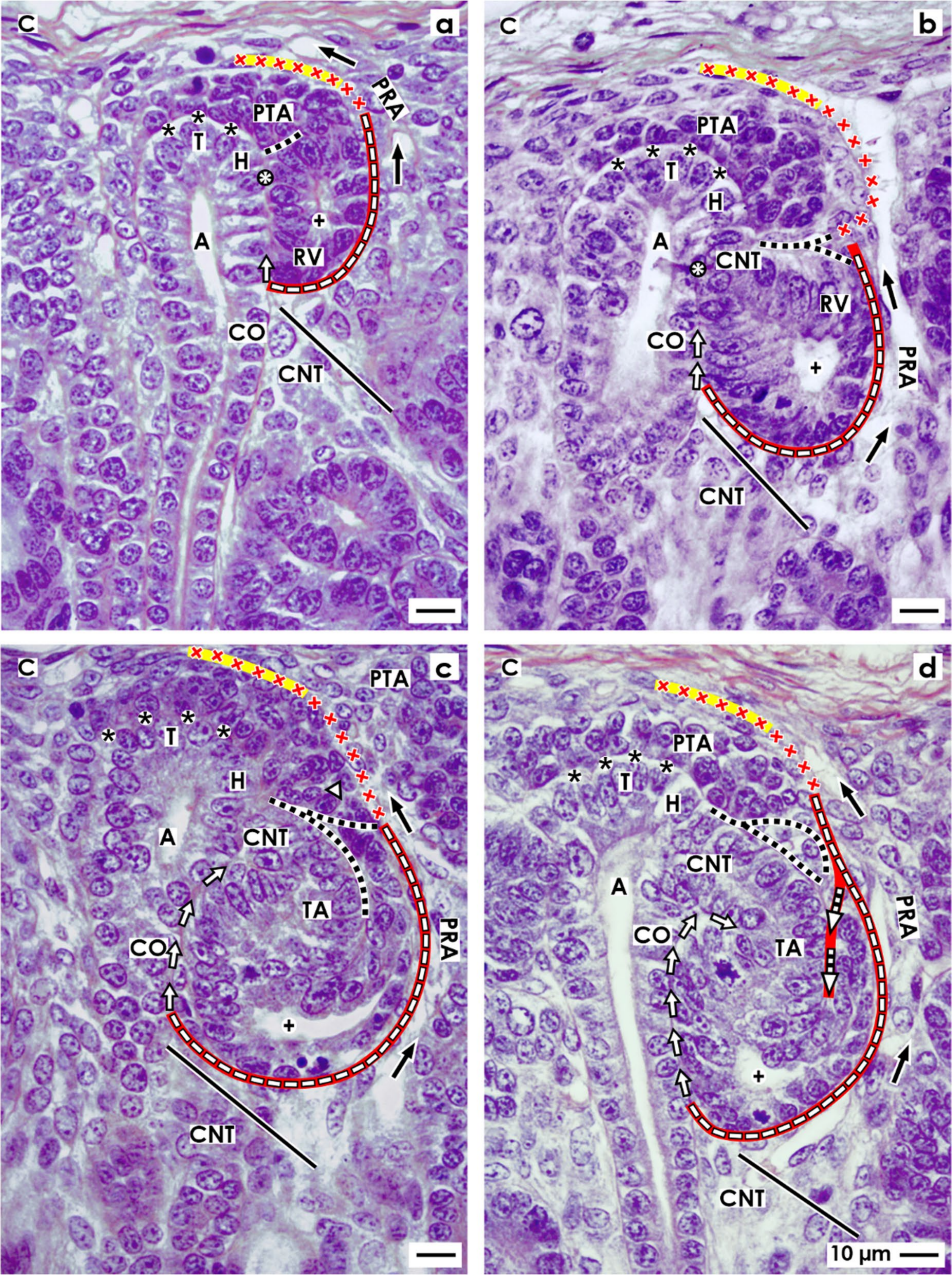
**a**–**d** View onto the interstitial situation at the **a** mature, **b** extending, **c** extended renal vesicles (RV), and the **d** early comma-shaped body (CSB) in the fetal human kidney during advanced pregnancy by the optical microscope. **a** While the mature RV arises, at its distal pole, a transverse but only partial separation (dashed black line) from the overlying pretubular aggregate (PTA) occurs. This is visible at the end of the clear interface (black asterisks) and the beginning of the adhesion (white asterisk) next to the section border between the head (H) and conus (CO) of the CD ampulla (A). The lateral part of the distal pole at the RV remains connected with the proximal end of the PTA via a two-layered progenitor cell strand (arrow head). **b** In the extending RV, the interjacent cleft transversely expands. The distal pole of the RV stays near the proximal end of the PTA so that a cone is formed by the subcapsular interstitium (yellow line with crosses) and the perivascular interstitium (white boxes with red line) of a perforating radiate artery (PRA). The future connecting tubule (CNT) starts to invade the CD ampulla, while the tubule anlage (TA) elongates. **c** Due to the vertical elongation of the CD ampulla and the internal folding in the extended RV, the interstitial cleft (white arrow) between the CNT and the progenitor cell strand turns direction from transverse to vertical, increases in length, and forms a pocket. In parallel, the narrow vertical interstitial cleft between the conus of the CD ampulla and the medial aspect of the renal vesicle expands in vertical direction. It originates from the cone, which is formed by the peritubular interstitium of a previously developed CNT (black line) and the perivascular interstitium of the perforating radiate artery. **d** While the early comma-shaped body is forming, the progenitor cell strand, which earlier connected the distal pole of the extended renal vesicle with the proximal end of the PTA, dissolves. This causes that the interstitial pocket yet fuses with the perivascular interstitium next to the PTA. As a consequence, between the tubule anlage and the developing lateral fold, a vertical cleft (white boxes with red line and arrow head) arises. This is a morphological sign that the glomerulus defoliates at the proximal pole. + Lumen

**Fig. 4:**
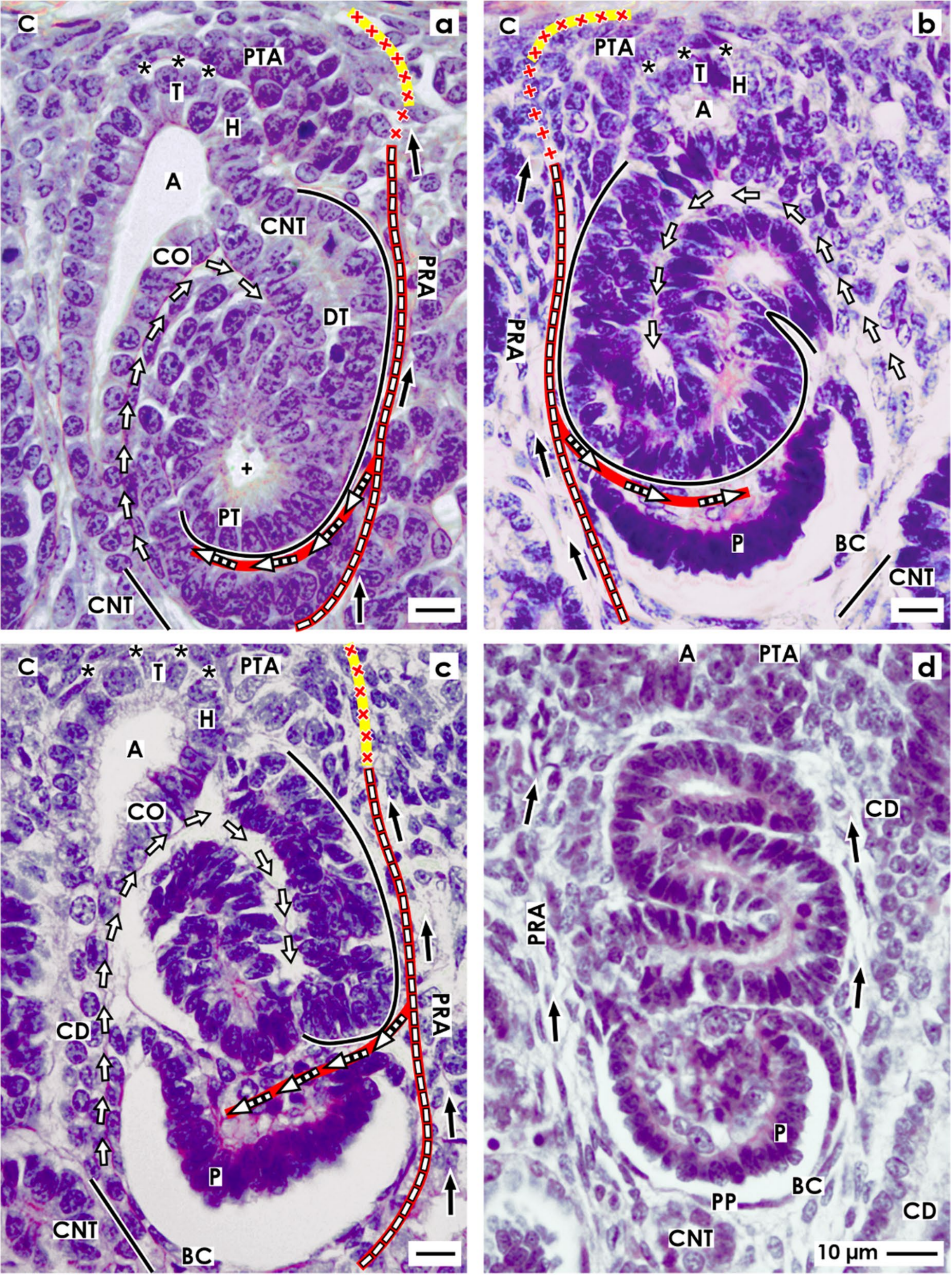
**a** – **d** View onto the interstitial situation at the **a** early, **b** mid, **c** late S-shaped body, and the **d** maturing nephron in the fetal human kidney during advanced pregnancy by the optical microscope. **a** In the early S-shaped body, its peritubular interstitium at the connecting tubule (CNT; black line) faces the proximal end of the overlying pretubular aggregate (PTA). The elongating distal tubule (DT) is exposed at the lateral aspect to the perivascular interstitium (white boxes with red line) of the perforating radiate artery (PRA, black arrows). At the deep lateral aspect, the arising glomerulus defoliates along a transverse cleft (white boxes with red line and arrow head), which is bordered by the forming proximal tubule (PT). Between the conus (CO) of the CD ampulla (A) and the medial aspect of the S-shaped body, a vertical but narrow interstitial cleft (white arrow) ascends up to the CNT. **b** In the mid S-shaped body, interstitial cells invade the transverse cleft at the deep lateral aspect. At the same time, as well as the afferent and efferent arterioles as the intra- and extraglomerular mesangium develop. Underneath, the developing glomerulus shows the visceral layer including the podocytes (P) and the parietal layer representing the Bowman’s capsule (BC). This is in touch with the peritubular interstitium of a previously developed CNT. At the medial aspect, the vertical interstitial cleft lines along the conus of the CD ampulla and the meandering tubule portions. In this case, the constellation is shown at the left side of a CD ampulla. **c** The late S-shaped body shows at its proximal pole the developing glomerulus. The Bowman’s capsule is becoming flat, the podocytes acquire a cobblestone-like appearance, and capillaries and the mesangium are forming. At the medial aspect, the S-shaped body is facing beside the CD ampulla and also the differentiating collecting duct (CD) tubule. The proximal pole is opposite the peritubular interstitium of the CNT of a previously formed nephron, at the lateral aspect by the perivascular interstitium of the PRA. The forming proximal, intermediate, distal, and connecting tubule portions become covered by a faint peritubular interstitium. **d** The maturing nephron is positioned vertically underneath the head of a CD ampulla. Within the expanding glomerulus beside the podocytes, also numerous capillaries and the mesangium are visible. The lateral aspects are opposite the perivascular interstitium of the ascending PRAs. The meandering tubule portions are surrounded by a faint peritubular interstitium. + Lumen, yellow line with crosses subcapsular interstitium

In the fetal human kidney during advanced pregnancy, the formation of new nephrons is restricted to the nephrogenic zone. Covered by the renal capsule (Fig. 1a–c), it consists of side-by-side aligned nephrogenic compartments (Table 1). In each of them, the initial development of a nephron can be observed. It starts in a determined sequence with the transient stages of nephron anlage and continues with the terminal differentiation of the maturing nephron in the subjacent maturation zone. When a transverse line is drawn at the section border between the head and conus of the neighboring collecting duct (CD) ampulla, a nephrogenic compartment is subdivided (Fig. 2a). In the upper district of progenitor cell recruitment, which is seen during the further development as a constant prone rectangle, the mesenchymal nephrogenic progenitor cells meet the epithelial progenitor cells, which are contained in the tip of the CD ampulla, at the nephrogenic niche. As a result of this interaction, first the pretubular aggregate arises, and then, the mesenchymal to epithelial transition takes place (Fig. 2b). This again announces the rising of the primitive renal vesicle (Fig. 2c and d). Due to the vertical elongation of the CD ampulla, the primitive renal vesicle remains in the area of nephron shaping, which is represented during the further development by an expanding quadrate. Here, the formation of the mature, extending, and extended renal vesicles (Fig. 3a–c) and the comma- (Fig. 3d) and S-shaped bodies (Fig. 4a–c) occurs. Then, in the subjacent maturation zone, the functional differentiation of the nephron proceeds (Fig. 4d).

When the arrangement of the interstitium is analyzed, beside the actual contours and the stage-specific location of the forming nephron, also the structural neighbors in a nephrogenic compartment have to be considered (Table 1). At the top, it is covered by the renal capsule (Fig. 1a–c), at the medial aspect by the ureteric bud derived CD ampulla, at the base by the connecting tubule of a previously developed nephron, and at the lateral aspect by a perforating radiate artery. Thus, the available space between the actual stage of nephron anlage and its structural neighbors reflects the current arrangement of the interstitium. However, during the running developmental process, not only the shape of the forming nephron but also the constellation with the structural neighbors is altering. For example, the forming nephron increases in size, takes progressive shapes, shifts between its structural partners, evolves proximal and distal poles, and shows asymmetrical forms at its medial and lateral aspects. In parallel, also the CD ampulla and the perforating radiate artery vertically elongate. Meanwhile, a network arises between the forming nephron and its structural neighbors, which contains, for example, collagen type 3 as a stabilizing compound of the extracellular matrix (Fig. 1b).

### Interstitium privatum in the renal capsule

As it was mentioned, the top of a nephrogenic compartment is covered by the renal capsule (Table 1, Fig. 1). It represents an organ-specific kind of the connective tissue. The capsula fibrosa is externally situated, consists of several strata, and protects the growing kidney against inappropriate influences. On microscopic specimens, one can see that numerous fibroblasts occur between the transversely lining layers of collagen bundles (Fig. 1a). Inside the renal capsule, numerous arterial, venous, and lymphatic vessels are visible. The vertically ascending perforating radiate arteries reach the inner side of the renal capsule for joining the Rete capillare capsulare (Fig. 3b). From the neonatal rabbit kidney, it is further known that the collagen in the capsula fibrosa is specifically stacked. Histochemical label revealed, for example, that the external bundles are not stained by tannic acid, while the internal bundles are intensely labeled (Fig. 1c).

The subjacent tunica muscularis contacts the nephrogenic zone. Here, numerous myofibroblasts and atypical and typical smooth muscle cells are noticed (Fig. 1a). Electron microscopy revealed that a part of these cells is covered by a distinct basal lamina, while others show only a faint glycocalyx (Fig. 1c). Further on, at the inner side of the renal capsule and next to the external part of the nephrogenic zone, the neighboring cells establish numerous contacts via cell projections so that the intra- and extracellular space between the cell projections represents a complex tunnel system (Fig. 1d and e). It may serve the transport and distribution of interstitial fluid and to keep the balance of fluid pressure between the renal capsule and the nephrogenic zone.

### Interstitial peculiarities between the renal capsule and the tip of a CD ampulla

Although it looks unspectacular in the optical microscope, a series of data indicates that the interstitial space between the inner side of the renal capsule and the tip of the CD ampulla is specially structured (Table 1; Fig. 1b and c).

The transverse mesenchymal cell layers: In the fetal human kidney, the distance between the tip of a CD ampulla and the inner side of the renal capsule is not accidental but has a vertical width of only 30 µm. Between them, the interstitial and nephrogenic progenitor cells are contained. It is striking that the innermost layer of the nephrogenic progenitor cells does not touch but is rather separated by a clear interface from the tip of the CD ampulla (Fig. 1a). At this specific site, the nephrogenic niche is visible.

The vertical microfibers: In the fetal human kidney and, for example, in the neonatal rabbit kidney, the distinct distance between the inner side of the renal capsule and the tip of a CD ampulla is caused by a special mounting. Immunohistochemical label for collagen type 3 in the neonatal rabbit kidney depicts extracellular microfibers. These originate at the inner side of the renal capsule and cross first the stromal and then the nephrogenic progenitor cell layers for the link with the basal lamina at the tip of a CD ampulla (Fig. 1b).

### Interstitial fronts at the niche, pretubular aggregate, mesenchymal-to-epithelial transition, and primitive renal vesicle

The meeting between the innermost layer of the nephrogenic mesenchymal progenitor cells and the epithelial progenitor cells contained in the tip of a CD ampulla indicates the precise coordinates of a nephrogenic niche (Figs. 1a and 2a). However, a special feature is that the bodies of the two kinds of progenitor cells do not touch. Instead, these are separated by the clear interface (Fig. 1c). For the kidney of neonatal rabbit, it was further demonstrated that the tip of a CD ampulla is covered by a striking basal lamina. Improved contrasting of specimens by tannic acid (Fig. 1d) or cupromeronic blue (Fig. 1e), for the purpose of transmission electron microscopical analysis, additionally showed that the extracellular matrix within the interface is composed by textured properties. Finally, a concise feature is that single projections of the nephrogenic mesenchymal progenitor cells are enveloped in a specific sleeve of extracellular matrix. By this construction, they vertically cross the interstitial interface to penetrate the basal lamina and to contact via tunneling nanotubes the basal plasma membrane of the epithelial progenitor cells, which are integrated in the tip of a CD ampulla.

After induction by morphogenic molecules, the nephrogenic mesenchymal progenitor cells, which are located at the niche, become angular, migrate, and aggregate first along the tip and then the head of the CD ampulla. As a result, the tear drop-like pretubular aggregate arises (Fig. 2a). It represents a specialized interstitial cell construct, which separates only in part from the overlying nephrogenic mesenchymal progenitor cell layer. While its thin distal end remains in contact with the overlying nephrogenic progenitor cells, its broad end further expands towards the connecting tubule of a previously developed nephron. Due to the typical shape and the distinct position of the pretubular aggregate, its interstitial surrounding is built up asymmetrically. Most of its lateral part is exposed to the subcapsular interstitium, which is located at the inner side of the renal capsule. In contrast, the clear interface is visible between the medial part of the pretubular aggregate and along the tip and head of the CD ampulla. Strikingly, the lateral part at the proximal end of the pretubular aggregate is positioned opposite the perivascular interstitium of a vertically lining perforating radiate artery. The mid of its proximal end faces the peritubular interstitium at the connecting tubule of a previously developed nephron. Determining for the future development, the medial part of its proximal end shows an adhesion at the section border between the head and conus of the CD ampulla.

Typical signs for the mesenchymal-to-epithelial transition (MET) are noticed only at the proximal end of the pretubular aggregate (Fig. 2b). The first polar cells are seen opposite to the cone, which is formed between the peritubular interstitium at the connecting tubule of a previously developed nephron and the perivascular interstitium at the perforating radiate artery. At the same time, the medial part at the proximal end of the pretubular aggregate expands the adhesion at the CD ampulla. This site reflects the future physiological connection between the connecting tubule of the presently forming nephron and the prospective collecting duct tubule.

The morphogenesis of the primitive renal vesicle is first recognized as an open clamp at the proximal end of the pretubular aggregate (Fig. 2c). A circle of polarized cells becomes visible, when the primitive renal vesicle is established (Fig. 2d). Although a small lumen is yet recognized, its distal pole remains connected with the overlying pretubular aggregate. In this situation, the lateral part of the pretubular aggregate is positioned near the subcapsular interstitium, while the lateral aspect of the primitive renal vesicle encounters the perivascular interstitium of the perforating radiate artery. Its proximal pole stays near the peritubular interstitium at the connecting tubule of a previously developed nephron. However, at the medial aspect of the primitive renal vesicle, the adhesion expands at the section border between the head and conus of the CD ampulla. As a consequence, the clear interface lining between the CD ampulla and the medial part of the pretubular aggregate is interrupted at the site of adhesion.

### Interstitial features at the mature, extending, and extended renal vesicles

Regarding the microanatomical situation, the subcapsular interstitium accompanies very early steps of nephron formation such as the recruitment of progenitor cells, the process of nephron induction, the formation of the pretubular aggregate, the mesenchymal to epithelial transition, and the arise of the primitive renal vesicle (Fig. 2c–d). However, while the CD ampulla elongates vertically, the primitive renal vesicle starts to separate at its distal pole from the overlying pretubular aggregate. At the same time, it is translocated from the district of progenitor cell recruitment to the subjacent area of nephron shaping. Henceforth, the installation at the medial aspect of the developing renal vesicle is bordered not anymore by the head but by the upper conus of the CD ampulla. At its lateral aspect, the renal vesicle faces yet the radially lining perforating radiate artery. Its distal pole is fixed near the proximal end of the overlying pretubular aggregate, while its proximal pole stays next to the connecting tubule of a previously developed nephron.

The development from the primitive to the mature renal vesicle coincides with a transverse but only partial separation from the pretubular aggregate. In parallel, the adhesion at the section border between the head and conus of the CD ampulla proceeds (Fig. 3a). The separation is first recognized as a narrow transverse cleft. It starts at the end of the clear interface and the initial site of adhesion, and it continues up to the center of the pretubular aggregate. At a later time, it can be observed that the arising cleft is invaded by interstitial cells. At the adhesion site, the connection between the future connecting tubule and the CD ampulla is under work. Surprisingly, the laterally located cells at the distal pole of the mature renal vesicle stay, for a moment, connected with the pretubular aggregate via a two-layered progenitor cell strand serving the progressive recruitment with progenitor cells. Initially, only a close adhesion is noticed between the medial aspect of the mature renal vesicle and the upper conus of the CD ampulla. Then, it is replaced by a narrow cleft, which expands vertically. This is formed due to a cone-shaped interstitial process, which originates in part from the perivascular interstitium of the perforating artery and in part from the peritubular interstitium at the connecting tubule of a previously formed nephron.

At the extending renal vesicle, one can observe that the future connecting tubule is perforating the epithelium of the CD ampulla at the border between its head and conus (Fig. 3b). Furthermore, it is registered that the cleft between its distal pole and the proximal end of the pretubular aggregate extends transversely. Especially remarkable, the lateral part of the distal pole at the extending renal vesicle is still connected with the overlying pretubular aggregate via the two-layered progenitor cell strand. The medial cell strand lines to the mid at the proximal end, while the lateral strand extends to the lateral part of the pretubular aggregate. It is noticed that the lateral cell strand is located at the perivascular interstitium of the perforating radiate artery. Further on, it crosses the subcapsular interstitium including the transverse progenitor cell layers to reach the inner side of the renal capsule. In parallel, between the upper conus of the CD ampulla and the medial aspect of the extending renal vesicle, an elongation of the narrow vertical interstitial cleft is recognized.

While the extended renal vesicle establishes, the tubule anlage expands vertically and laterally (Fig. 3c). At its distal end, the epithelium of the future connecting tubule is connected with the CD ampulla. In the lumen near the proximal pole, the inner epithelial fold becomes visible. This causes the interstitial cleft, which is seen between the tubule anlage and the laterally located progenitor cell strand, to elongate first in an oblique and then in a vertical direction. The entire lateral aspect of the extended renal vesicle is exposed to the perivascular interstitium of the perforating radiate artery. The proximal pole of the extended renal vesicle faces the peritubular interstitium at the connecting tubule of a previously developed nephron. From here, the interstitium invades as a narrow vertical cleft between the conus of the CD ampulla and the medial aspect of the extended renal vesicle.

### Interstitial constellation at the comma-shaped body

A special but to date unexplored meaning has the interstitium at the developing comma-shaped body (Fig. 3d). In this phase, beside the elongation of the tubule anlage and the extension of the epithelial folds, the proximal–distal positioning and the defoliation of the arising glomerulus can be observed. Due to the appearance of new contours, parts of the surrounding interstitium disappear, while others retreat or are rebuilt. Interestingly, the progenitor cell strand at the distal pole dissolves, so that the connection between the comma-shaped body and the pretubular aggregate is lost. As a result, the interstitial cleft, located between the tubule anlage and the progenitor cell strand, is fused with the perivascular interstitium of the neighboring perforating radiate artery. At the lateral aspect of the comma-shaped body, precisely between the lateral fold and the tubule anlage, a vertical interstitial cleft develops. It ends at the turn up of the inner fold. This is a clear morphological sign that the glomerulus is starting to defoliate. The future Bowman’s capsule (lateral leg of the lateral fold) faces the perivascular interstitium of the perforating radiate artery. The proximal pole is now located opposite the peritubular interstitium at the connecting tubule of a previously developed nephron. Between the conus of the CD ampulla and the medial aspect of the comma-shaped body, the narrow vertical interstitial cleft further expands. At the connection site between the future connecting tubule and the conus of the CD ampulla, it changes direction to follow the peritubular interstitium at the developing tubule portions towards the geometric center of the comma-shaped body.

### Interstitial characteristics at the S-shaped body

During the formation of the early S-shaped body, the physiological link between the connecting tubule and the CD ampulla is completed (Fig. 4a). At this site, an interstitial cone becomes visible. It is bordered at the top by the proximal end of the overlying pretubular and the subcapsular interstitium and by the laterally situated perivascular interstitium of the perforating radiate artery and the peritubular interstitium of the presently forming connecting tubule. Between the conus of the CD ampulla and the medial aspect of the S-shaped body, the narrow vertical interstitial cleft is extended. It lines up to the peritubular interstitium, which surrounds inside the S-shaped body the elongating connecting, distal, intermediate, and proximal tubule portions. At its lateral aspect, the peritubular interstitium of the extending distal and intermediate tubule portions is fused with the perivascular interstitium of the perforating radiate artery. Importantly, the transverse cleft further prolongs at the deep lateral aspect. It opens up between the overlying proximal tubule portion and the subjacent visceral podocyte cell layer of the arising glomerulus. Moreover, one can observe that the cells, which originate from the perivascular interstitium of the perforating radiate artery, invade the transverse cleft. This is a morphological sign that the capillary loops and the intra- and the extraglomerular mesangium are starting to develop. The proximal pole of the S-shaped body is represented by the developing Bowman’s capsule. It is facing at its medial part the peritubular interstitium of a previously developed connecting tubule and at its lateral part the perivascular interstitium of the perforating radiate artery.

During development of the mid S-shaped body, at its proximal pole, the Bowman’s capsule and the Bowman’s space expand. Furthermore, the podocytes develop a typical cobblestone-like appearance (Fig. 4b). In the overlying transverse cleft, beside the numerous capillaries, the formation of the intraglomerular mesangium and the arise of the afferent and efferent arterioles are recognized. At the opening of the transverse cleft, the close topological relation between the perivascular interstitium of the perforating radiate artery and the glomerular tuft is obvious. At the medial aspect of the S-shaped body, it is noticed that a certain distance is formed between the conus of the CD ampulla and the tubule portions. The interjacent space is filled by a loose peritubular interstitium.

### Interstitial transition from the nephrogenic zone to the maturation zone

The late S-shaped body as the last transient stage of nephron anlage stays positioned perpendicular to the renal capsule. It demonstrates a morphological segmentation along its proximal–distal axis (Fig. 4c). At the proximal pole, the typical morphological features of the glomerulus appear. For example, the Bowman’s capsule is becoming flat, the Bowman’s space is clearing, and the podocytes show now their typical shape. Further numerous glomerular capillaries become visible, while a broadening of the intraglomerular mesangium occurs. At the medial aspect, the Bowman’s capsule is located near the conus and neck of the CD ampulla and also along the differentiating collecting duct tubule. However, the elongating tubule portions are separated from the neck and conus of the CD ampulla by a newly formed but faint peritubular interstitium. At the lateral aspect, the situation is different, since the connecting and distal tubule portions face the perivascular interstitium of the perforating radiate artery.

Due to the ongoing radial extension of the nephrogenic zone, the late S-shaped body is leaving the area of nephron shaping for the further development in the maturation zone (Fig. 4d). Its distal pole, which is seen at the tip head of the CD ampulla, is positioned near the subcapsular interstitium. At the proximal pole and in its interior, the arising glomerulus and the meandering tubule portions are covered by a faint peritubular interstitium. In this region, single strands of extracellular matrix are noticed, which line in vertical direction. At the meandering tubule portions, one can recognize that within their coves, a filigree interstitium arises. Occasionally, long thin cells with an extended but flat nucleus are here noticed. These are similar to the telocytes.

## Discussion

### Interstitial features of the outer renal cortex

Covered by the renal capsule, the interstitium of the outer renal cortex in the fetal kidney during advanced pregnancy is located between the parenchymal structures of the arising nephrons and the developing collecting duct tubules [21]. It consists of a network of organo-typical extracellular matrix, in particular microfibers, proteoglycans, and other special glycoproteins [22–24]. At this stage, distinctly different and far matured progenitor cells such as fibroblasts, fibrocytes, myofibroblasts, dendritic cells, macrophages, lymphocytes, and granulocytes are integrated [25]. These are surrounded by the interstitial fluid, which contains electrolytes, nutrition, metabolites, respiratory gas, waste, hormones, cytokines, growth factors, and morphogenic molecules. Additionally, important parts of the interstitium are the arterial, venous, and lymphatic vessels and the site-specific innervation [26]. In contrast to the adult kidney, the low vascularization in the outer cortex of the fetal human kidney during advanced pregnancy suggests a major role for hypoxic physiological conditions during the process of nephron formation [27].

The interstitium in the renal cortex of the adult kidney was earlier divided into the peritubular and periarterial interstitium and into the glomerular and extraglomerular mesangium [28]. However, due to the complexity of the developing structures, the interstitial configuration in the outer cortex of the fetal human kidney during advanced pregnancy is not comparable. Recent publications and the here shown microscopic images (Figs. 1, 2, 3 and 4) depict that the contained interstitium is heterogenous, and that it is facing not only the covering renal capsule [18, 21, 24, 29] but also the underlying pool of progenitor cells, the transiently developing stages of nephron anlage, the maturing nephrons, the elongating collecting duct (CD) ampullae, and the differentiating collecting duct tubules [30–32].

### Interstitial source at the developing nephron

A lesser-known but very special interstitial configuration is noticed between the inner side of the renal capsule and the tip of the CD ampulla. The space available has an average width of only 30 µm (Fig. 1a) [33]. Although it is small and looks not particularly spectacular, it was demonstrated that the here occurring interstitial and nephrogenic progenitor cells are stored, sorted, and promoted for the prospective development [34, 35]. Immunohistochemistry revealed that along the inner side of the renal capsule, one layer of stromal cells is positioned, while underneath one to two layers of nephrogenic progenitor cells occur. Although the innermost layer faces the tip of the CD ampulla, it is separated by a striking interstitial interface (Fig. 1c–e). At the niche, the transversely lining progenitor cell layers are crossed by vertically lining extracellular microfibers. These can be labeled, for example, by soybean lectin or antibodies recognizing collagen type 3 as a typical molecular compound of the local extracellular matrix (Fig. 1b) [22, 36]. For the neonatal rabbit kidney, it was further shown that the vertical microfibers are both static and under conversion, since the extracellular matrix-stabilizing tissue transglutaminase 2 (Tgase2) and the matrix-degrading metalloproteinase 9 (MMP9) are co-expressed at this site [37]. Especially remarkable are the ultrastructural features of the interface at the tip of a CD ampulla (Fig. 1c–e) [18, 38–40] and the here expressed extracellular matrix glycoproteins [41]. Especially interesting are the projections of the nephrogenic mesenchymal cells, which cross the interstitial matrix within individual sleeves to contact the basal aspect of the epithelial progenitor cells contained in the tip of a CD ampulla [42, 43]. Undeniably, the observed features collectively indicate a highly specialized interstitial microenvironment, which provides the driving support for the initial process of nephron formation [44].

### Translocation between interstitial workbenches

**Fig. 5:**
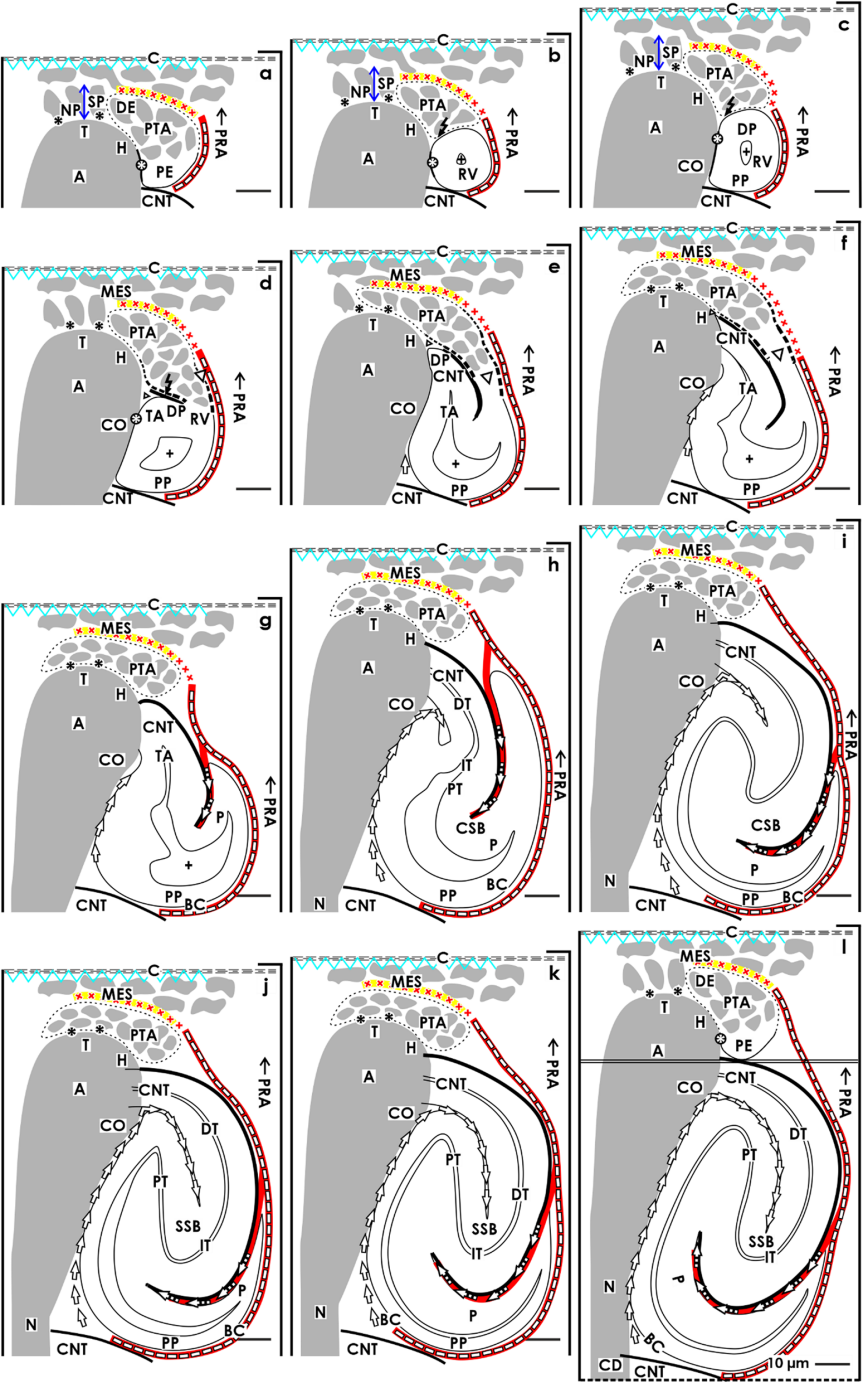
a–l Sketches almost true to scale illustrate the interstitial situation at the developing nephron in the fetal human kidney during advanced pregnancy. Depicted are the configuration of the interstitium at the **a** pretubular aggregate (PTA), the **b** site of the mesenchymal-to-epithelial transition, and the **c** primitive renal vesicle (RV). Inserts, C renal capsule, SP stromal progenitor cells, NP nephrogenic progenitor cells, DE distal end, PE proximal end, PTA pretubular aggregate, PRA/black arrow perforating radiate artery, black asterisks interface, white asterisk adhesion, CNT connecting tubule of a previously developed nephron, A collecting duct (CD) ampulla, T tip, H head, CO conus, N neck, flash partial separation of RV from PTA, + lumen, C renal capsule, NZ nephrogenic zone, MZ maturation zone. Demonstrated is further the extension of the interstitium at the **d** mature, **e** extending, and **f** extended renal vesicles (RV). DP distal pole, PP proximal pole, TA tubule anlage with the future connecting tubule (CNT) of the presently developing nephron, arrow head progenitor cell strand between RV and PTA. Shown is also the interstitial configuration at the **g** early, **h** mid, and (**i**) late comma-shaped bodies (CSB). DT distal tubule, IT intermediate tubule, PT proximal tubule, P future podocytes, BC presumptive Bowman’s capsule. Finally, depicted is the extension of the interstitium at the **j** early, **k** mid, and **l** late S-shaped bodies (SSB) including the defoliation of the glomerulus at the proximal pole and the physiological connection of the CNT with the CD ampulla. Light blue/zigzag, interstitium within the renal capsule. Dark blue/vertical double arrow, interstitium between the inner side of the renal capsule and the tip of a CD ampulla. Yellow line with crosses, subcapsular interstitium. White boxes with red line, perivascular interstitium at the perforating radiate artery. Black line, peritubular interstitium at the connecting tubule. White boxes with red line and arrowhead, interstitial cleft at the deep lateral aspect between the tubule anlage and the defoliating glomerulus during formation of the comma- and S-shaped bodies. Dashed black line, oblique interstitial cleft between the tubule anlage and the progenitor cell strand, which connects the distal pole of the renal vesicles with the proximal end of the pretubular aggregate. White arrow, vertical narrow interstitial cleft between the CD ampulla and the medial aspect of the shaping nephron

The data depicted in Fig. 5 and Tables 1 and 2 demonstrates that the interstitium at a developing nephron is not centered but distributed among various sites. For this to occur, the requirement is that the expanding interstitium adapts and also alters in accordance to the altering shapes of the nephron and its neighboring structures. Additionally, based on the developmental progress observed, a spatial shifting between the different structures has taken place. This can be registered first by the appearance of the nephrogenic niche, by the aggregation of the pretubular aggregate, mesenchymal to epithelial transition, and then by the formation of the primitive renal vesicle (Figs. 2a–c and 5a–c). These stages are restricted to the district of progenitor cell recruitment. Especially remarkable is the fact that they are always first positioned next to the tip and then to the head of the CD ampulla.

However, due to the partial separation of the primitive renal vesicle, the following process of translocation produces a change in the backdrop. Therefore, the mature, extending, and extended renal vesicles (Figs. 3a–c and 5d–f) and comma- (Figs. 3d and 5g–i) S-shaped bodies (Figs. 4a–c and 5j–l) develop in the underlying area of nephron shaping. Thereby, these stages are facing the elongating conus of the CD ampulla. Lastly, the maturing nephron is positioned in the subjacent maturation zone next to the differentiating collecting duct tubule (Fig. 4d). Recent investigations additionally demonstrated that each of the listed stages exhibits a different shape, is recognized within personal coordinates, and is facing individual parts of the structural neighbors [12, 19, 20]. This again shows that each stage of nephron anlage is exposed to a typical claim of the interjacent interstitium, which can exhibit existing, evolving, transient, degrading, or lasting properties (Fig. 5; Tables 1 and 2).

### Narrow interstitial cleft at the medial aspect of the developing nephron

From the kidneys of preterm and low birth babies, it is known that noxae provoke the reduction in width of the nephrogenic zone [6]. Although it has not been closely investigated, one must assume that the loss of tissue concerns not only the stages of nephron anlage but also the structural neighbors such as the CD ampullae, the sprouting microvessels, the establishing interstitium, and probably also the renal capsule [12]. As a consequence, it is highly important to develop a deeper understanding of these details, when the interstitium is seen as an instance that masters the formation of a nephron or when this process is harmed by the noxae impairing nephrogenesis [4, 14, 21, 24].

Particularly striking is the narrow interstitial relation between the CD ampulla and the medial aspect of the developing nephron. At the tip and head of the CD ampulla, the nephrogenic progenitor cells and the medial part of the pretubular aggregate face the clear interface (Figs. 1a, 2, and 5a and b) [18]. While the primitive renal vesicle develops, the interface is interrupted by its adhesion at the section border between the head and conus of the CD ampulla. Recent findings from different groups have shown that this serves the primary linking with the future connecting tubule [45–47]. During the ongoing development, the mature, extending, and extended renal vesicles (Figs. 3a–c and 5c–f) face only the upper conus of the CD ampulla, while the comma- (Figs. 3d and 5g–i) and S-shaped bodies (Figs. 4a–c and 5j–l) are additionally situated at the lower conus and neck and even at the upper part of the differentiating collecting duct tubule. Most interestingly, in parallel to the formation of the nephron, a steady elongation of the interjacent interstitial cleft is observed. However, not only the vertical course of the narrow interstitial cleft is impressive but also its arrangement between nearly congruent surfaces. These are provided mainly by the conus of the CD ampulla and the medial aspect of the actual stage of nephron anlage. For the neonatal rabbit kidney, it was demonstrated that the composition of the here situated fibrous extracellular matrix is not homogenous but differs along the various sectors at the CD ampulla [48]. It appears that the narrow interstitial cleft is part of a slide bearing, which is best suited for the short and targeted exchange of morphogenic proteins.

### Open interstitial flank at the lateral aspect of the developing nephron

Completely different is the interstitial situation at the distal pole, lateral aspect, and proximal pole of the forming nephron. While the renal vesicles arise, the contained tubule anlage elongates so that finally the inner epithelial fold becomes visible (Fig. 3a–c). This causes that the interstitial invasion at the transverse cleft, which is seen between the distal pole of the renal vesicle and the proximal end of the pretubular aggregate, changes direction to become an oblique interstitial pocket. When the comma-shaped body is forming (Figs. 3d and 5g), the progenitor cell strand is dissolving so that the earlier connection between the distal pole of the renal vesicles and the pretubular aggregate is lost (Figs. 3c and 5d–f). As a consequence, the interstitium within the oblique pocket is fused with both the subcapsular interstitium and the perivascular interstitium of the neighboring perforating radiate artery. Thus, the entire lateral aspect of the S-shaped body (Figs. 4a–c and 5j–l) and of the maturing nephron (Fig. 4d) is henceforth exposed to the perivascular interstitium of the perforating radiate artery. Regarding this special structural relation, it is most likely that a punctual disturbance of these perivascular interstitial cells [49], which invade the S-shaped body at its lateral aspect, may cause the earlier described pathological damage on the developing glomeruli such as the shrunken glomerular tuft and the expanded Bowman’s capsule [7]. In the future, it is of great importance to understand whether the depicted interstitial constellation is the subject of an integrating or a mastering function [50].

## Conclusions

The interstitium at the developing nephron in the fetal human kidney during advanced pregnancy is a fundamental but largely unexplored part of the nephrogenic zone. This work sheds light and provides the first microanatomical insights reported up to date. The analysis indicates that the interstitium at the forming nephron has a complex structure, which consists of existing, transient, stage-specific, and differently far matured compartments. Precise explanations, clear illustrations, and scaled sketches inform about the respective position. Thus, the created structural basis enables henceforth to investigate the subject in detail. In particular, the search for early traces left by noxae impairing nephrogenesis in preterm and low birth weight babies is supported.

## Data Availability

The data sets generated and/or analyzed during the current study are not publicly available due to ongoing research but are available from the corresponding author on reasonable request.
